# Multi-center study on mortality in children, and adults with sickle cell anemia-risk factors and causes of death

**DOI:** 10.1038/s41598-024-58328-9

**Published:** 2024-04-13

**Authors:** Salam Alkindi, Salma Al-Jadidi, Safa Al-Adawi, Refaat Abdullah Elsadek, Ali Al Madhani, Maryam Al-Nabhani, Anil V. Pathare

**Affiliations:** 1 Department of Haematology, College of Medicine and Health Sciences, Sultan Qaboos University, P. O. Box 35, 123 Muscat, Oman; 2 Department of Medicine, Nizwa Hospital, Nizwa, Oman; 3 Department of Medicine, Sohar Hospital, Sohar, Oman; 4 Emergency Department, Royal Hospital, Muscat, Oman

**Keywords:** Sickle cell disease, Predictors of mortality, Acute chest syndrome, Sepsis, Sudden death, SCD, Diseases, Health care, Medical research, Risk factors

## Abstract

Sickle cell disease (SCD) is a major public health burden worldwide with increasing morbidity and mortality. The study evaluates the risk factors associated with mortality in SCD patients, between the years 2006 and 2020 at three hospitals in Oman. The analysis includes clinical manifestations, haematological, biochemical, and radiological parameters, use of antibiotics, and blood and exchange transfusions. Our cohort included 123 patients (82 males, 41 females), with a median age of 27 (Interquartile Range 21–35 years). SCD related complications included acute chest syndrome (ACS) in 52.8%, splenic sequestration in 21.1%, right upper quadrant syndrome in 19.5%, more than > 6 VOC/year in 17.9%, and stroke in 13.8%. At the terminal admission, patients had cough, reduced O_2_ saturation, crepitation and fever in 24.4%, 49.6%, 53.6% and 68.3% respectively. Abnormal chest X-ray and chest CT scan were seen in 57.7%, and 76.4% respectively. Laboratory parameters showed a significant drop in hemoglobin (Hb) and platelet counts from baseline, with a significant rise in WBC, LDH and CRP from baseline (*p* < 0.05, Wilcoxon Signed Ranks test). All patients received antibiotics, whereas, 95.9% and 93.5% received simple blood transfusions, and exchange transfusions respectively, and 66.6% required non-invasive ventilation. Among the causes of death, ACS is seen in 32 (26%), sepsis in 49 (40%), and miscellaneous in 42 (34%). Sudden death was seen in 32 (26%) of patients. Male gender, with low HbF, rapid drop in Hb and platelet, and increased in WBC, LDH, ferritin, and CRP, correlated significantly with mortality in this cohort.

## Introduction

Sickle cell disease (SCD) is one of the commonest monogenic diseases with highest prevalence in sun-saharan Africa (500–2000/100.000)^1^. It is caused by a single amino acid mutation, where glutamic acid is replaced by valine in the beta chain of hemoglobin molecule^2,3^. The hemoglobin-S polymerizes in post-capillary venules, causing the red blood cells to assume a distorted, sickle-like shape.

Sickle cell disease is a major public health problem in Oman with a marked increase in morbidity and mortality. The estimated sickle cell gene frequency is between 5.8%, and 5.1% with the prevalence of sickle cell trait and SCD is 4.8% and 0.3% respectively^4,5^.

Sickle cell disease is characterized by repetitive episodes of vaso-occlusion causing painful episodes, hemolytic anemia, and increased risk of infections impacting severely on survival. Survival estimates however, have continued to improve, with over 95% of SCD patients reaching adulthood, at least in the developed world^6^. In 1994, Platt reported the median survival for patients with HbSS/Sβ^0^ thalassemia to be 42–48 years for males and females respectively^7^. This has increased to 53 and 58 years in Jamaica by 2001^8^, and 58 years in the United States in 2014^9^. In recent years, patients are reported to live into their eighth decade^10^. This improvement is attributed to the widespread use of newborn screening programs, penicillin prophylaxis, the universal vaccination program, and the establishment of a comprehensive clinical care program (CCCP) with Hydroxyurea, and blood transfusions^11^.

Although survival rate for children and adults with SCD has improved, however there are still patients who are lost prematurely^11–15^. There are many causes of death including sepsis, painful episodes precipitating acute chest syndrome (ACS), and stroke. Previous studies have attempted at identifying risk factors for death including the development of dactylitis, co-inheritance of alpha thalassemia, high Hb F, low baseline Hb, and high WBC count as predictors of severe adverse events^13,14^. Some of these were substantiated in subsequent studies, and others were not. The data on specific causes and risk factors for mortality in our country are not available. In a study by Tawfic et al.^16^, ACS was the main cause of ICU admission in patients with SCD. Further, the use of inotropic support and/or mechanical ventilation was an indicator of a high mortality rate among these patients. In another study in children with SCD, Jaiyesimi and Kasem^17^ showed that ACS was common irrespective of SCD severity, and all patients appeared to be at risk, but it was increased by Vaso-occlusive crisis (VOC).

Although ACS has remained a leading cause of death, there are no established risk factors for death in patients with SCD in Oman. We are presenting death from three different hospitals in Oman, to identify the main causes of death and its associated risk factors.

## Materials and methods

In this retrospective study, 123 SCD patients, who died between 2006 and 2020 at three hospitals in Oman were enrolled, after written approval from the Medical Research Ethics Committee (MREC # 1322), College of Medicine & Health Sciences. All methods were carried out in accordance with relevant guidelines and regulations of the Institution where this research was carried out. Due to the retrospective nature of the study, the need for informed consent was waived by the Medical Research Ethics Committee (MREC #1322). The inclusion criteria included SCD patients of any age, who died between 2006 and 2020. The data was obtained from the electronic patient record system and included demography, the season during death, previous SCD manifestations such as the frequency of painful crisis, ACS, splenic and hepatic sequestration, dactylitis, and stroke. We also analyzed the hematological, biochemical, and radiological parameters at baseline (stable during Outpatient visit), and in the terminal event, as well as the use of antibiotics, ventilatory support, and use of blood /exchange transfusions.

The laboratory results were obtained at baseline and at the terminal event. Hemoglobin and platelet counts were obtained at baseline and nadir, whereas, reticulocyte counts, and white blood cell count (WBC) were recorded at baseline and their maximum. Biochemical parameters included C-reactive protein (CRP), Liver function tests, and Serum LDH and were collected on arrival and at maximum during the terminal event. Additionally, data on basal serum ferritin and HbF were also collected.

### Definitions of parameters and SCD complications

To classify the time of death, the year was divided into two seasons based on a mean temperature of 33 °C, between April and August, and a mean temperature of 25 °C between September and March. Stroke was defined as acute neurologic syndrome due to vascular occlusion or hemorrhage in which neurologic symptoms or signs lasted more than 24 h. ACS was defined as the presence of new pulmonary infiltrates on a chest X-ray film, CT scan of the chest, or both, in association with acute respiratory tract symptoms. Sudden death was defined as death from any cause happening within 24 h after hospital admission. Acute splenic sequestration was defined as a decrease from baseline in the hemoglobin level or hematocrit of at least 20 percent, plus a simultaneous increase in the size of the spleen to at least 2 cm below the left costal margin. Right upper quadrant syndrome was defined as SCD painful crisis with signs and symptoms affecting the liver or gallbladder.

Clinical symptoms recorded included temperature, respiratory symptoms, and O_2_ saturation by pulse oximetry. Radiology studies including chest X-ray, CT scan and ultra sound of abdomen. Further, SCD therapeutic management protocols like non-invasive ventilatory support, antibiotics, blood transfusion and blood exchange were also recorded. Terminal events were defined as the last hospital admission leading to death of the patient. Causes of death were classified to ACS (clinical & radiological signs), sepsis (clinical, and laboratory evidence with positive microbial culture) and miscellaneous (all others). Miscellaneous causes include cardiac events, pulmonary embolism, stroke, RTA and malignancy.

### Statistical analysis

The statistical package for social science (IBM SPSS, USA ver.23, Armonk, NY) was used to analyze the collected data. Normally distributed data were characterized as mean with standard deviation, whereas, data that was not normally distributed was characterized as median with interquartile range (IQR) for continuous variables and percentage and frequency for categorical variables. Wilcoxon Signed Ranks test was used to test the significance of the association between subgroups for various clinical and laboratory parameters. An alpha of < 0.05 was considered to be the statistically significant *p* value.

## Results

123 patients (82 males, 41 females) who were enrolled in the study had a median age (IQR) of 27 (21–35) years, with a range between 1.5 to 79 years. Painful VOC episodes > 6/year were seen in almost one-fifth of this cohort (17.9%), while most patients had a significant past history of SCD-related complications including ACS (52.8%), splenic sequestration (21.1%), right upper quadrant syndrome (19.5%) and stroke (13.8%) (Table 1). At the terminal event, fever, cough, abnormal findings in chest examination, and reduced O_2_ saturation (pulse oximetry) were seen in 68.3%, 24.4%, 53.6%, and 49.6% respectively.

**Table 1:** Demographic, clinical and laboratory parameters in SCD cohort (n = 123).

Parameters		Cases	*p* value
Demography			
Median age in years (IQR)		27 (21–35)	
Gender, Total cases, n, M: F		82:41	
Median age in years, males (IQR)		26.5 (20.75–33)	NS
Median age in years, females (IQR)		28 (23–39)	
Past history			
H/o VOC episodes			
n (%) (< 3/yr)a		78 (63.4)a	
n (%) (3–5/yr)b,		23 (18.7)b	
N (%) (> 6/yr) c,		22 (17.9)c	
H/o ACS, n (%)		65 (52.8)	
H/o stroke/neurological syndrome, n (%)		17 (13.8)	
H/o splenic sequestration, n (%)		26 (21.1)	
H/o right upper quadrant syndrome, n (%)		24 (19.5)	
Symptoms and signs			
Fever, n (%)		84 (68.3)	
Cough, n (%)		30 (24.4)	
Crepitation, n (%)		67 (54.5)	
Tachycardia, n (%)		97 (78.8)	
Hypotension, n (%)		42 (34.1)	
Tachypnea, n (%)		38 (30.9)	
Reduced O_2_ saturation, n (%)		61 (49.6)	
Abnormal chest X-rays, n (%)		71 (57.7)	
Abnormal CT scans, n (%) [n = 95]		74 (77.8)	
Abdominal ultrasound, %		54 (43.9)	

Laboratory features	Basal	Nadir/Max/Terminal	
Median (IQR) hemoglobin, g/L	9.5 (8.6–10.7)	6.8 (5.5–7.7)	< 0.05^#^
Median (IQR) WBC counts, 10^9^/L	11 (8.7–13)	21 (15–30.8)	< 0.05^#^
Median (IQR) platelet counts basal, × 10^9^/L	319 (201–442)	80 (41–139)	< 0.05^#^
Median (IQR) retic count, %		5.75 (3.9–8.5)	
Median (IQR) HbF, %		7.7 (3.6–15)	
Median (IQR) HbF ≥ 8.5%		15.7 (13–20.4)	< 0.05^#^
Median (IQR) HbF < 8.5%		4.9 (2.6–7.6)	
Median (IQR) S.LDH, u/L	652 (425–1362)	1616 (750–4213)	< 0.05^#^
Median (IQR) CRP,	43 (15.5–144)	171 (54–325.5)	< 0.05^#^
Median (IQR) *S. bilirubin*, mg/dl	32 (11)	105 (46–260.5)	< 0.05^#^
Median (IQR) serum ferritin (ng/L)		1064 (323–2987)	
Interventions			
Antibiotics, n (%)		123 (100)	
Simple blood transfusions, n (%)		118 (95.9)	
Exchange blood transfusions, n (%)		115 (93.5)	
NIV/Ventilation, n (%)		82 (66.6)	

^#^, Wilcoxon Signed Ranks Test; IQR, Interquartile range; NS, Not significant; M, Male; F, Female; H/o, History of; VOC, Vaso-occlusive crisis; ACS, Acute chest syndrome; SLDH, Serum lactate dehydrogenase; CRP, C reactive protein; NIV, Non-invasive ventilation.

Amongst the haematology parameters, there was a significant drop in the median hemoglobin and platelet counts from baseline, with a significant rise in the WBC counts (*p* < 0.05, Wilcoxon signed ranks test). The median level of baseline hemoglobin (g/dl) dropped significantly from 9.5 to 6.8 g/dl. Reticulocytes were elevated at 5.75%. The median level of white blood cell count (× 10^9^/L) increased significantly to 21 from a baseline level of 11 (*p* < 0.05). The median platelet count (× 10^9^/L) dropped significantly to 80 from a baseline level of 319 (*p* < 0.05). Amongst the biochemical parameters, the median CRP levels (mg/L) significantly increased from 43 at arrival to the hospital, to the median CRP max value of 171 (*p* < 0.05). Similarly, the median serum bilirubin levels (mg/dl) rose significantly from 34 at admission to 105 (*p* < 0.05). Further the median serum LDH levels (U/L) also significantly increased from 652 at admission to 1616 at the time of the terminal event (*p* < 0.05).

Chest X-ray was abnormal in 71 (57.7%) patients, whereas, the ultrasound showed abnormalities among 54 patients (43.9%). Although a CT scan was done only in 94 patients, it was abnormal in 84 (89.3%) patients. All these patients received antibiotics, whereas, simple and exchange blood transfusions were given in 118 (95.9%) and 115 (93.5%) of patients respectively. Further, 82 patients (66.6%) required ventilatory support.

At the terminal event, 32 (26%) patients died within 24 h of admission to the hospital. 49 (40%) died with sepsis, 32 (26%) died due to ACS associated with multi-organ failure, while the remaining 42 (34%) died due to miscellaneous causes including cardiac events, pulmonary embolism, stroke, RTA, and some died at home. (Table 2) Mortality was significantly correlated with a cutoff of basal Hb, basal WBC count, and HbF% of 7 gm/dl, 15 × 10^9^/L, and 8.5% respectively. Leukocytosis was seen in a small number of patients at baseline, but it was significantly high at the time of death. In addition, HbF was a strong predictor of death in all groups. The majority of patients 89 (72.4%) who died had a HbF < 8.5%. About 30% of all the patients, had baseline thrombocytosis, however many of the patients had thrombocytopenia at the time of death. Splenectomy (surgical or auto-splenectomy) was seen in about 50% of all the groups, but no significant differences between all causes of death.

**Table 2:** Causes of death and impact of selected factors on death with inter-group analysis.

Parameters/terminal event	ACS n, (%)	*p* value	Sepsis n, (%)	*p* value	Miscellaneous n, (%)	*p* value	Overall causes n, (%)	*p* value
Total	32 (26)		49 (40)		42 (34)		123 (100)	
Median age (Yr)	29.5		24		30		27	NS
Splenectomy/autosplenectomy, n	15 (46.9)		21 (42.9)		21 (50)		58 (47.1)	NS
Basal hemoglobin ≥ 7 g/L,	32 (100)	*p* < 0.000^#^	47 (96)	*p* < 0.000^#^	37 (88.1)	*p* < 0.0003^#^	116 (94.3)	*p* < 0.000^#^
Basal hemoglobin < 7 g/L,	0		2 (4)		5 (11.9)		7 (5.7)	
Hemoglobin Nadir ≥ 7 g/L,	14 (43.7)	*p* = 0.5	17 (34.7)	*p* = 0.07	20 (47.6)	*p* = 0.8	51 (41.5)	*p* = 0.12
Hemoglobin Nadir < 7 g/L,	18 (56.3)		32 (65.3)		22 (52.4)		72 (58.5)	
WBC counts basal, ≥ 15 × 10^9^/L,	3 (9.4)	*p* = 0.0003^#^	5 (10.2)	*p* < 0.0003^#^	5 (11.9)	*p* = 0.001^#^	13 (10.6)	*p* < 0.000^#^
WBC counts Max, ≥ 15 × 10^9^/L,	25 (78.1)		38 (77.6)		26 (61.9)		89 (72.3)	
Hb F% ≥ 8.5,	7 (21.9)	*p* = 0.008^#^	16 (32.7)	*p* = 0.04^#^	11 (26.2)	*p* = 0.01^#^	34 (27.6)	*p* < 0.0003^#^
Hb F% < 8.5,	25 (78.1)		33 (67.3)		31 (73.8)		89 (72.4)	
Basal platelets ≥ 400 × 10^9^/L	10 (31.2)	*p* = 0.08	17 (34.7)	*p* = 0.14	17 (40.5)	*p* = 0.8	44 (35.8)	*p* = 0.04^#^
Nadir platelets < 100 × 10^9^/L	22 (68.8)		29 (59.2)		19 (45.2)		70 (56.9)	
Culture positive, n	8 (25)		36 (73.5)		10 (23.8)		54 (43.9)	NS
Sep–March mean tem ≈25.5	23 (71.8)	*p* = 0.04^#^	32 (65)	*p* = 0.078	18 (43.9)	*p* = 0.5	44 (47.3)	*p* = 0.65
April–Aug-mean temp ≈ 33	9 (18.2)		17 (35)		23 (56.1)		49 (52.7)	
Sudden death ≤ 24 h	11 (34.4)		14 (28.6)		7 (16.6)		32 (100)	NS

ACS, Acute chest syndrome; #, Wilcoxon Signed Ranks Test.

**Figure 1:**
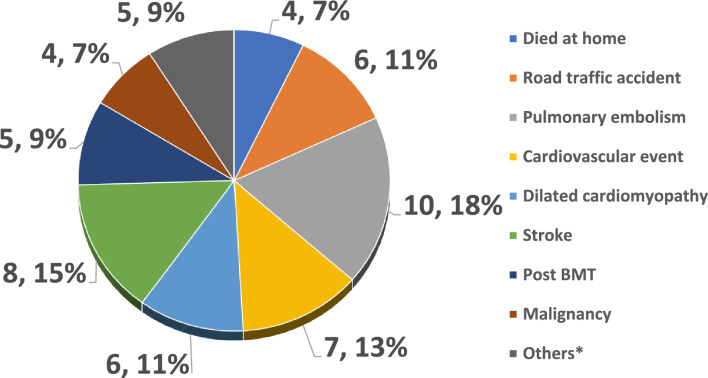
Details of miscellaneous causes (n, %).

In addition, death due to ACS was significantly seen in the colder months of the year, (*p* = 0.04), and sudden death was more likely to happen in ACS, and sepsis group. When we looked at miscellaneous causes of death, cardiac events were most frequent, followed by pulmonary embolism, and stroke. There were a few cases, who died at home, RTA, or cancer (Fig. 1).

Table 3 shows a comparison of the various risk factors affecting mortality from a literature review. It shows that although survival has improved, patients still die prematurely, especially males; and in this study, the overall proportion of death among males was double that seen in females (66% vs 33%) although the gender ratio in the general SCD Omani patients is 0.51^19^.

**Table 3:** Comparative study of Literature data on parameters influencing mortality in SCD patients.

Country/parameters	Oman Present study	Turkey^19^	Saudi Arabia^21^	Brazil^22^	Brazil^23^	UK^24^	USA^25^
No. of deaths (%)	123	45 (6.1)	77 (0.73)	281 (16.77)	6924 (70.5)	9 (19.6)	16,654 (65)
Sex (Male:female)	82:41	27:18	43:34		3372:3552	5:4	
Age at death in Males, (Mean ± SD) Range in years	(28.1 + 12.4) 3–79	(34.1 ± 10) 18–54	(30 ± 14) 16–67	(32.71 ± 14.25) 1–68	29.4 ± 19.6	(32.6 + 12.3) 19–50	33.4 (95%CI 33–33.7)
Age at death in Females, (Mean ± SD) Range in years	(31.7 + 13.2) 2–66	40.1 ± 15 17–64	(27 ± 13) 14–67	(33.85 ± 14.8) 3–75	33.3 ± 20.3	(34.25 + 13.2) 26–43	36.9 (95%CI 36.5–37.4
Terminal events ACS, n (%)	32 (26)	8 (28.5)	22 (28.5)	71 (25.27)	1322 (19.1)	3 (33)	–
Infection, n (%)	49 (40)	–	17 (21.9)	82 (29.2)	3859 (55.7)	–	2 (18.1)
Death with 24 h, n (%)	32 (26)	–	–	26 (9.25)	728 (10.5)	–	2 (18.1)

Amongst 67 cases with sepsis, 53 (79.1%) were culture-proven, with 11 (20.7%) patients showing multiple microorganisms, while bacterial, fungal, and viral microorganisms were isolated in 32 (60.4%), 6 (11.3%), and 4 (7.5%) respectively (Table 4). Among the positive cultures, gram-negative organisms were the commonest, and the highest was seen in the sepsis group, with an increasing number of fungal and viral illnesses. Also, it’s important to note that 16.5% of the cohort had serological evidence of viral hepatitis B, C, and E.

**Table 4:** Culture proven and confirmed infections at time of death.

Total cases with sepsis, n (%)		67 (100)
Culture proven microorganisms, n (%)		54 (79.1)
No growth, n (%)		13 (20.9)
Multiple organism, n (%)		14 (26.4)
Bacteria, n (%)		50
	*Salmonella* Spp., n (%)	16 (32)
	Pseudomonas	6 (12)
	Others	28 (56)
Viruses, n (%)		13
	Parvo virus	4 (30.7)
	Others	9 (69.3)
Fungi, n (%)		14
	Candida Albicans	4 (28.6)
	Candida Krusei	4 (28.6)
	Other species	6 (42.8)
Viral hepatitis, n (%)	HBV, HCV, HEV	20 (16.5)*

*HBV* Hepatitis B virus, *HCV* Hepatitis C virus, *HEV* Hepatitis E virus.

*Of total patients.

## Discussion

Although survival has improved in SCD, patients still die prematurely, especially males; and in this retrospective study, the overall proportion of death among males was double that seen in females. Similarly, the median age in males was lower (26.5 v/s 28 years), as compared to the females, but this difference was not statistically significant. Comparable findings were also reported in other studies from the region and beyond^14,15,18,20,21^.

Karacaoglu et al.^20^, from Turkey, demonstrated that there were more deaths among males, than females, and the mean age of death in their female population was 40.1 years. Although patients are still dying young, data from a French SCD cohort in children under the age of 5 years showed that there is an improvement in survival attributed to the establishment of what is known as complete comprehensive patient care for SCD^21^. Al-Suliman et al.^22^ in a study on adults from the eastern province in Saudi Arabia reported a higher prevalence of males 55.8% versus females 44.2% female, with the mean age of the male patients being 30 ± 14 years (range, 16–67 years) and of the female patients 27 ± 13 years (range, 14–67 years). The first Brazilian single institutional study in 2017 showed that the mortality rate was 18.87% among adult SCD patients^23^. Further, combined data between 1997 and 2017, from Brazil showed that mortality was more in the males (50.4%), and patients aged between 25 and 34 years had a higher incidence of deaths^24^. In another study from Brazil between 2000 and 2018, during the entire period the mean ages at death is significantly lower for males, than for females, being 29.4 (± 19.6) versus 33.3 (± 20.3) years respectively^25^. In a study from the UK, the CCU mortality for SCD patients was 19.6% between 2000 and 2007, with the mean ages for males and females respectively being 32.6 and 34.25 years^26^. Further, in a study from the USA with data spanning over 27 years between 1979 and 2005, the mean age at death was significantly different for males (33.4 years) than for females (36.9 years)^27^. Nonetheless, in recent years, patients are reported to live into their eighth decade as reported by Ballas^10^ and indeed in this study cohort, we had a SCD patient who died at the age of 79.

Significantly VOC (> 6 episodes/year) were seen in about 20% of cases, reflecting that recurrent VOC remains a marker of severe disease, and these patients accumulate disease-related end-organ damage that leads to their mortality^28^. However, about two-thirds of patients in this cohort (63.4%) had relatively mild disease, indicating the need for intense vigilance in picking up alarm signs for early mortality in some of these relatively mild disease cases.

Many of our patients had a history of ACS, stroke, hepatic & splenic sequestration as well as dactylitis, which were shown to be independent predictors of early childhood mortality in SCD patients^7–9,20^. Sixty-five (52.8%) patients had a prior history of ACS, one of the major complications of SCD, and contributed to the terminal event in 25(20.3%) of this cohort. Furthermore, 84 (68.3%) patients presented with fever, cough (24.4%), and 61(49.6%) had reduced O_2_ saturation, while 67 (54.5%) had abnormal findings in the chest examination in the terminal episode, with 82 (66.6%) needing ventilatory support. Similarly, 71 (57.7%) patients in this cohort had an abnormal chest X-ray, which was one of the defining features of ACS, and 77.8% had an abnormal CT scan of the chest. Importantly, Knight-Madden et al.^29^, and Tawfiq et al.^16^ have shown that mechanical ventilation was found to predict mortality and increased utilization of hospital resources. In this cohort 66.6% needed ventilator assistance, the majority of these patients received simple blood transfusions [118 (95.9%)] and many needed exchange transfusions [115 (93.5%)] as well.

Sepsis seems to be a major contributor to the terminal event as seen in 49 (40%) of cases in this cohort. These patients were characterized by a significantly high CRP, and LDH along with a significant rise in the WBC counts from the baseline, and a significant drop in platelets^30^. Among patients with sepsis, almost 80% of these patients had positive microbial cultures confirming sepsis. These patients received multiple broad-spectrum antibiotics during their terminal event. In the cooperative study of SCD from the USA, only a few bacteria were detected in lung samples at bronchoscopy, possibly since bronchoscopy was performed after empiric antibiotic administration, which is a universal practice^31^. Furthermore, even among SCD patients who had an autopsy done on them, sepsis was identified as the leading cause of death in a postmortem study^32^. More recent paper by Ballas indicated that sepsis contributed to death in a significant number of patients with SCD^33^.

Among the miscellaneous causes, multiorgan failure with sudden death including cardiac events with dilated cardiomayopathy seem to be a leading cause, although pulmonary embolism, stroke and RTA were also other significant causes of mortality in this cohort. Sudden death with bone marrow fat emboli seems to be associated with HbSC and HbSβ^**+**^ Thalassaemia or the milder SCD phenotypes^34^. However, in this SCD patient cohort HbSβ^**+**^ Thalassaemia was seen in 22.5% with no HbSC patient and 75.5% patients having HbSS genotype. This is similar to findings from other studies^15,18^. Similarly pulmonary embolism (PE), stroke, and RTA were also significant causes of mortality in this cohort. PE is the leading presentation of thromboembolic complications in SCD^35,36^, and stroke prevalence was relatively low in our population (probably due to the high prevalence of alpha thalassemia, Alkindi et al.^5^); however it remains as an important cause of morality. RTA contributed to about 5% of overall total deaths, although no data is available if it was linked to opioid use while driving, on not^33^. Among the miscellaneous also few patients had Bone marrow transplant with GVHD and died with sepsis, and also few patients had malignancy including acute myeloid leukemia, multiple myeloma, sarcoma and myelodysplasia.

Laboratory data showed that there was a statistically significant drop in levels of hemoglobin and platelets count at the terminal event in this study cohort (Table 1). A low hemoglobin level has been previously shown to correlate with an increased risk of death with stroke^18^. Although the majority of patients had a baseline Hb > 7 g/dl, the median Hb fell from 9.5 to 6.8, during the terminal event, in all causes of death (Table 2). On the contrary, literature reports show that a higher hemoglobin (Hb) level correlated with an increased risk of acute chest syndrome and painful crisis^18^. This was also seen in this study, using the cutoff basal Hb > 7 g/dl and basal WBC > 15 × 10^9^/L (Table 2). Consistent with this, many patients in this cohort needed either simple transfusion (95.9%) or exchange transfusions (93.5%).

In a previous prospective study by Plat et al.^7^, the most straightforward laboratory risk factor related to the cause of death was fetal hemoglobin level. Patients with high levels had an improved life expectancy, while adults who had low levels of fetal hemoglobin as children were likely to die earlier than those who had high levels^7^. Thus, when using a cutoff of HbF of 8.5, there was a clear protective effect of high HbF against all types of death, with a significant proportion of these deaths occurring in the low HbF group, (*p* < 0.05, Wilcoxon signed ranks test, Table 1). This may reflect the heterogeneity of HbS-associated haplotypes in Oman. Although the Arab-Indian haplotype is common in the Eastern provinces in Saudi Arabia^22^ in Oman, it only constitutes 25% in the SCD patient population, while Benin and Bantu haplotypes form 50% and 25% respectively^37^. Another, confounding factor related to HbF is hydroxyurea use, which is seen in 36% of this current SCD cohort.

An elevated WBC count was an independent predictor of disease severity as seen in our study. Quinn and Miller^38^ in a prospective study reported that leukocytes are known to be involved in the process of vaso-occlusion, and leukocytosis in adults is associated with an increased frequency of ACS and death^39^. Our patients also showed a significant rise in the WBC counts in the terminal event and using a cutoff of 15 × 10^9^/L, it was shown that there was a statistically significant rise in the WBC counts with a median WBC count of 24 and IQR between 20 and 33 (Table 1) and this correlated significantly with sepsis during the terminal event.

High serum ferritin was thought to be one of the associated features of mortality among patients with SCD, reflecting both iron overload, as well as inflammatory status. Increased gastrointestinal absorption of iron has been reported in sickle cell disease, because of the associated chronic hemolysis, and anemia. In our study cohort, the median serum ferritin (ng/L) was 1064 with IQR between 323 and 2987. Interestingly, Akinbami et al.^40^, recently reported that 90% of subjects with sickle cell disease had normal iron stores.

Elevated CRP can occur in SCD, during steady state, and in crisis, as reported by us and Okocha et al.^41,42^. It represents an underlying inflammatory/ infective, process or tissue necrosis and showed a statistically significant rise, in our cohort, especially among patients who died of sepsis/ multi-organ failure (Table 1). In our cohort, the basal median CRP was high (43 mg/L) and showed a statistically significant rise in the terminal episode with a median of 171 (*p* < 0.05). Among 67 cases with sepsis, 54 (79.1%) were culture-proven, with 11 (20.7%) patients, showing multiple microorganisms, while bacterial, fungal, and viral microorganisms were isolated in 32 (60.4%), 6 (11.3%), and in 4 (7.5%) respectively. The most frequent organisms were gram-negative organisms, similar to the recent report of the rising gram-negative organisms, reflecting an increasing use of external lines and acquisition of hospital acquired infections^43^.

Our study demonstrated a high serum LDH, high serum bilirubin, and high CRP that further increased significantly toward the terminal event. Several studies have shown these observations and they indicate the degree of inflammation, tissue necrosis, infarction, and haemolysis that is accentuated during the terminal phase^29,44,45^. Our study demonstrated a statistically significant rise in these parameters in the terminal episode substantiating the above (Table 1). These factors with the associated leukocytosis, thrombocytopenia, and drop in hemoglobin heralded the onset of multiorgan failure in this cohort.

The cause of death was established by the course of illness during hospitalization, supportive laboratory evidence and telephonic enquiries with relatives in case of out of hospital deaths of these patients. Although ACS has remained a leading cause of death, there are no established risk factors for death in patients with SCD in Oman. In a study from Oman by Tawfic et al.^16^ ACS was the main cause of ICU admission in patients with SCD. Further, the use of inotropic support and/or mechanical ventilation was an indicator of high mortality rate among these patients. Jaiyesimi and Kasem^17^ showed that ACS was common irrespective of SCD severity in children from Oman, and all patients appeared to be at risk, but it was increased in patients with vaso-occlusive crisis.

When we looked at the time of death, we observed increased mortality due to ACS and sepsis during the colder months of the year (September to March). This is not unusual as viral infections such as respiratory syncytial virus and seasonal influenza predominate during that period and it is known to be one of the leading viral causes of ACS. Our own data and others showed similar patterns^46,47^. We also observed that over 30% of deaths occurred within 24 h of hospital admission. This data is also similar to the previous studies and the highest numbers were reported in the ACS and sepsis groups^48^. In the absence of post-mortem studies, (which are rarely accepted by the patient’s relatives), the exact cause will remain elusive or putative, although the recent literature is suggestive of increased cardiovascular events in this situation.

SCD is a complex condition, with life-threatening complications that interfere with the patient’s normal life owing to repeated painful episodes, and the associated multi-system involvement. Although this is a retrospective study, however, it identified that ACS and sepsis were the most important preventable risk factor associated with mortality in this SCD patient cohort. A high index of suspicion and vigilance is required for patients presenting with fever, tachycardia, and signs of ACS, associated with a drop in Hb, with raised WBC, CRP, LDH, and ferritin, to identify these patients early and initiate appropriate rapid management protocols.

In summary, the ability to identify the risk factors that are associated with increased mortality among SCD patients permits accurate prognostication and provides effective prophylactic management strategies. This study shows that male gender, low HbF, substantial drop in hemoglobin and platelet, as well as increased in WBC counts, serum LDH, ferritin and CRP, correlated significantly with mortality risk during the terminal event in patients with SCD.

## Data Availability

Data is available on request from the authors. The corresponding author can be contacted for details of the study.
